# Hungry for Change: The Experiences of People with PKU, and Their Caregivers, When Eating Out

**DOI:** 10.3390/nu14030626

**Published:** 2022-01-31

**Authors:** Grace Poole, Alex Pinto, Sharon Evans, Suzanne Ford, Mike O’Driscoll, Sharon Buckley, Catherine Ashmore, Anne Daly, Anita MacDonald

**Affiliations:** 1 Faculty of Health, Education & Life Sciences, Birmingham City University City South Campus, Westbourne Road, Edgbaston, Birmingham B15 3TN, UK; grace.poole@mail.bcu.ac.uk; 2 Birmingham Women’s and Children’s NHS Foundation Trust, Steelhouse Lane, Birmingham B4 6NH, UK; alex.pinto@nhs.net (A.P.); sharon.morris6@nhs.net (S.E.); catherine.ashmore@nhs.net (C.A.); a.daly3@nhs.net (A.D.); 3 National Society for Phenylketonuria, P.O. Box 6046, Sheffield S12 9ET, UK; suzanne.ford@nspku.org; 4 North Bristol NHS Trust, Southmead Road, Bristol BS10 5NB, UK; 5 School of Health and Education, Middlesex University, Room WG41A (Williams Building), The Burroughs Hendon, London NW4 4BT, UK; m.odriscoll@mdx.ac.uk; 6 Department of Psychology, Faculty of Health, Psychology and Social Care, Manchester Campus, Manchester Metropolitan University, 53 Bonsall Street, Manchester M15 6GX, UK; sharon.j.bucley@stu.mmu.ac.uk

**Keywords:** phenylketonuria, eating out, low protein food, restaurants

## Abstract

For patients with phenylketonuria (PKU), stringent dietary management is demanding and eating out may pose many challenges. Often, there is little awareness about special dietary requirements within the hospitality sector. This study’s aim was to investigate the experiences and behaviours of people with PKU and their caregivers when dining out. We also sought to identify common problems in order to improve their experiences when eating outside the home. Individuals with PKU or their caregivers residing in the UK were invited to complete a cross-sectional online survey that collected both qualitative and quantitative data about their experiences when eating out. Data were available from 254 questionnaire respondents (136 caregivers or patients with PKU < 18 years and 118 patients with PKU ≥ 18 years (*n* = 100) or their caregivers (*n* = 18)). Fifty-eight per cent dined out once per month or less (*n* = 147/254) and the biggest barrier to more frequent dining was ‘limited choice of suitable low-protein foods’ (90%, *n* = 184/204), followed by ‘no information about the protein content of foods’ (67%, *n* = 137/204). Sixty-nine per cent (*n* = 176/254) rated their dining experience as less than satisfactory. Respondents ranked restaurant employees’ knowledge of the PKU diet as very poor with an overall median rating of 1.6 (on a scale of 1 for extremely poor to 10 for extremely good). Forty-four per cent (*n* = 110/252) of respondents said that restaurants had refused to prepare alternative suitable foods; 44% (*n* = 110/252) were not allowed to eat their own prepared food in a restaurant, and 46% (*n* = 115/252) reported that restaurants had refused to cook special low-protein foods. Forty per cent (*n* = 101/254) of respondents felt anxious before entering restaurants. People with PKU commonly experienced discrimination in restaurants, with hospitality staff failing to support their dietary needs, frequently using allergy laws and concerns about cross-contamination as a reason not to provide suitable food options. It is important that restaurant staff receive training regarding low-protein diets, offer more low-protein options, provide protein analysis information on all menu items, and be more flexible in their approach to cooking low-protein foods supplied by the person with PKU. This may help people with PKU enjoy safe meals when dining out and socialising with others.

## 1. Introduction

Eating out, defined as eating foods that are prepared by others and consumed out of the home in food establishments such as restaurants, cafes, canteens, and fast-food outlets, is a growing trend. It is a well-established core social activity among people in the UK [1,2]. Eating similar foods is a cue for social connection, providing an avenue for people to communicate and relate to each other and many people prefer to gather to share a meal rather than eat alone [3,4]. People with phenylketonuria (PKU), an inherited metabolic disorder, characterised by the inability to hydrolyse the amino acid phenylalanine, are treated with a low-phenylalanine and aspartame-free diet. Whilst this dietary treatment is critical to avoid neurological damage, it is complex, with the natural protein intake of patients with classical PKU being decreased to as low as 20% of regular intake when prescribed dietary treatment only. Eating outside the home may be uncomfortable for people with PKU as they must constantly navigate social situations in which they are unable to eat what others eat, with most of the regular meal items being excluded. 

There is an expectation in society that people can eat out at any time, any place, anywhere. Food and drinks are at the heart of consumer culture, increasing the pressure and desire on people with PKU to eat outside the home. According to the Kantar Worldpanel survey, in 2018, 98% of people in the UK reported eating or drinking ‘out’, with overall UK expenditure on food and drink reaching £49 billion a year [5]. Also in 2018, in an English survey of 2241 people aged 16 years and over, 68% had eaten in a restaurant in the last month, while 41% had eaten in a pub, bar or nightclub. Restaurants, takeaway food and cafes or coffee shops were the most popular options for eating out in the UK [6]. The Office for National Statistics (ONS) (2019) estimated that a UK household spent on average £38.80/week on food prepared out of the home, including £18.60 on restaurants and cafés. In a Food Standards Survey (2018), 85% of respondents ate out for dinner, 70% for lunch and 38% for breakfast; this was more common among young people (aged 16–34 years) and men tended to eat out more than women for breakfast, lunch and dinner. 

Eating out in restaurants presents many challenges for individuals with PKU. Menu choices in restaurants usually do not state what ingredients are added to dishes or give their protein content, leaving a person with PKU the difficult choice of non-participation or choosing inappropriate foods, intensifying dietary adherence issues that may lead to poor metabolic control. They may lack self-confidence skills to seek the necessary help to secure appropriate food choices. Although there is legislation (The Food Information Regulations 2014 (“FIR”) [7] and The Food Information (Amendment) (England) Regulations 2019) [8] requiring all operators to disclose food allergens, there is no mandatory catering training for special dietary provision. Evidence suggests that there are significant knowledge gaps regarding special diets among the employees of the UK hospitality industry [9,10,11]. The workforce in restaurants often consists of young employees, some of whom are undertaking their first job, and there may be high employee turnover with low engagement. When training is initiated, it is usually for new employees and there may be infrequent training updates [10]. 

Therefore, it is important to explore factors that contribute towards experiences of people with PKU when eating out. This will help to characterise the main issues encountered, any social impacts and the effect on their ability to follow their dietary treatment. Thus, this study was designed to investigate the experiences of patients with PKU, and their caregivers, in eating establishments. The aim was to identify common problems of eating out in order to improve their dining experiences in the future. 

## 2. Materials and Methods

### 2.1. Methods

This was a cross-sectional study using an online survey that collected both qualitative and quantitative data from adults with PKU and caregivers of children and adults. Respondents were excluded if they did not reside in the UK. 

The questionnaire was built in the Online Surveys platform (https://www.onlinesurveys.ac.uk, accessed on the 2 November 2020) to gather quantitative data. This was placed on the UK National Society for Phenylketonuria (NSPKU) website, with additional promotion on the NSPKU Twitter, Instagram and Facebook pages. The questionnaire was open for 7 months, from April until October 2020.

### 2.2. Questionnaire

The non-validated questionnaire contained 20 questions (Table S1). Eight questions were multiple choice, *n* = 8 multiple responses, *n* = 2 Likert scale and *n* = 2 open ended questions. Thirteen questions invited additional comments. 

The questionnaire was developed by dietitians with expert practical and scientific knowledge of PKU (AP, SE, CA, AD, AM), a colleague from the NSPKU (SF), a researcher (MO) and a student dietitian from Birmingham City University (GP). It was reviewed by colleagues and lay people to ensure its readability and then amended according to feedback.

### 2.3. Data Collected

The questionnaire was divided into three sections, collecting information on patient age, frequency of eating out, factors that prevented the individual from eating out, impact of low protein diet, factors that affected the choice of restaurant, and influences that affected meal choice in restaurants. Information on the perception of knowledge about a low-protein diet by restaurant staff, descriptions, and characteristics of good restaurants for patients with PKU, and opinion of restaurant chains was also requested. All data collected were based on the patients/caregiver’s experiences when eating out.

### 2.4. Statistics

Quantitative data analysis (inferential and descriptive statistics) was carried out with the Statistical Package for the Social Sciences (SPSS) version 25 (SPSS Inc., Chicago, IL, USA). For multiple response questions, only descriptive statistics were used (inferential statistics are not normally used with such questions). For testing differences between two categorical variables, chi square was used. Statistical significance was set at *p* < 0.05.

Qualitative data analyses of open-ended responses were carried out in NVIVO version 12 PRO (QSR International Pty Ltd.). The whole survey dataset was imported into NVIVO so that the coding of open-ended responses could be broken down by survey questions. All open-ended questions responses were analysed thematically.

### 2.5. Ethics

Ethical approval was obtained from the Birmingham City University ethics committee prior to commencement of the study (Poole/6128/R(A)/2020/Mar/HELS FAEC: What knowledge and attitudes do restaurateurs have about provision of the phenylketonuria (PKU) diet?/What are the experiences of people with PKU, and their caregivers, when eating out in restaurants or cafes?). At the beginning of the online questionnaire, respondents gave consent, and it was emphasised that the questionnaire completion was voluntary. Potential respondents were advised that data from the survey may be published in an anonymized form. If names or hospitals were mentioned in verbatim abstracts these were removed from results presented in this manuscript.

## 3. Results

Data were available from 254 participants (whole or partial completions of the questionnaire). The number of respondents for each question varied, as not all respondents answered all questions. Fifty-four per cent (*n* = 136/254) of responses were related to people with PKU under 18 years of age. Forty-six per cent (*n* = 118/254) of responses were from people aged ≥18 years of age 100 adults with PKU and 18 caregivers of adults with PKU aged ≥18 years. 

### 3.1. Frequency of Dining Out

Most respondents of the questionnaire dined out only once per month or less (*n* = 147/254; 58%). Eighteen per cent (*n* = 46/254) reported doing so ‘once per week’, 18% (*n* = 45/254) said they did so ‘once per fortnight’, and 6% (*n* = 15/254) did so ‘2–3 times per week.’ Furthermore, most participants (*n* = 204; 80%) expressed the desire to dine out more often; and reported factors which prevent this (Table 1). The biggest barrier overall was ‘limited choice of suitable low protein foods’ (90%, *n* = 184/204) followed by ‘no information about the protein content of foods’ (67%, *n* = 137/204). More adults with PKU (*n* = 27, 30%) said they ‘Have no choice but to eat foods that are not permitted in the PKU diet’ compared to the responses of children’s caregivers (*n* = 12, 10%). More caregivers of children compared with adults with PKU described issues such as ‘restaurants refusing to prepare low protein foods they provided’ e.g., pasta (41%, *n* = 47 children vs. 33%, *n* = 29 adults); ‘feeling hungry after eating out due to limited food choice’ (34%, *n* = 39 children vs. 24%, *n* = 21 adults); and ‘no information about the protein content of foods (72%, *n* = 83 children vs. 60%, *n* = 53).

Twenty-two responses answered “other”. Several responses indicated that the cost of dining out was higher or of poor value for people with PKU e.g., ‘often it ends up costing quite a lot of money for what is actually eaten’. They said there was more wasted food, or they provided low-protein ingredients for the restaurant to cook without a price reduction or they had to pay more than they received if sharing the bill with people who do not have PKU. Other issues identified by respondents included: ‘if no information is provided about the food’s protein content, I tend to go over my daily allowance and suffer migraines and I do not feel 100% the next day;’ and ‘I will not ask for low-protein food to be cooked, as too many people are within earshot. Usually, staff taking orders are very young’.

### 3.2. Choice of Restaurant

Eighty-nine per cent (*n* = 227/254) said the choice of restaurant was influenced by the need to follow a low-protein diet for the person with PKU. Factors that influenced the choice of restaurant are given in Table 2. Parents of children < 18 years of age were more likely to choose a restaurant if ‘catering staff were happy to cook with low-protein foods’, (46%, *n* = 63 vs. 34%, *n* = 40 of those aged ≥18 years). Parents of children < 18 years of age were less likely than adults with PKU to say ‘Like to socialise with friends/family regardless of food choice’ (*n* = 30, 22% vs. adults *n* = 50, 42%), and ‘good choice of low protein foods on the menu’ (parents of children aged <18 years: *n* = 93, 68% vs. adults: *n* = 93, 79%).

Respondents added 17 verbatim comments describing factors that influenced their restaurant choice. These included: ‘My daughter goes to places she’s tried before just so she has the information she needs about protein content in food’; ‘she will always Google the menu to see if there is anything on the menu, if nothing available she will make an excuse to her friends to decline going’. Other comments included: ‘there are limited places to go and even then, the same food is eaten every time’; and ‘the majority of restaurants will not cook food I supply for my 5-year-old daughter so we can’t go very far’.

### 3.3. Practices When Eating Out

Seventy-four per cent (*n* = 188/254) of respondents said that they ordered from the menu and chose something that may be suitable for PKU. Respondents for children under 18 years of age were more likely than adults with PKU to bring in some low protein food from home and ask the restaurant/cafe to cook it or to prepare an alternative meal (Table 3). Differences by age were statistically significant (*p* < 0.001). There were 20 other comments about food choices when eating out which included: ‘we usually feed our child with PKU before going out and then choose either chips or olives in the restaurant’; ‘I call ahead to discuss suitable food choices’; and ‘I do a combination of ordering low-protein options, taking low-protein bread with me, sometimes pasta too’.

### 3.4. Views on Restaurant Brands

Respondents rated a series of popular chain restaurants regarding the suitability of meal choices and the customer services they received to help them with their dietary needs. The scale ran from ‘very poor’ to ‘very good.’ The results are summarised in Table 4. Only one restaurant scored more than 50% of ratings as good or very good (Hungry Horse, 53%, *n* = 82/154). Many high street chain restaurants had less than 25% of users saying they were good or very good at helping provide suitable food or supporting patients with PKU. 

### 3.5. Overall Satisfaction When Eating Out

The overall dining experience was unsatisfactory for most respondents. The median overall satisfaction rating was 4 (*n* = 254) (on a scale of 1 for extremely poor to 10 for extremely good). Sixty-nine per cent (*n* = 176/254) of respondents rated overall satisfaction as 5 or less.

### 3.6. Rating of Restaurant/Café Employee Staff Knowledge about Phenylketonuria (PKU)

Knowledge of PKU and dietary management was rated as very poor by respondents with an overall median rating of 1.6 from 254 responses (on a scale of 1 for extremely poor to 10 for extremely good). There were 100 free text comments to this question from which the themes given in Table 5 were derived.

### 3.7. Helpfulness of Restaurants/Cafes in Finding a Solution to Cater for PKU

Sixty-three per cent (*n* = 159/254) of respondents said that they had at least one positive experience when dining out, particularly at local/ independent restaurants and non-chain restaurants (‘after repeated visits, they went out of their way to cater for PKU’) and it was considered particularly helpful when restaurants provided a full list of ingredients with their protein content. However, only one third of respondents (33%, *n* = 83/254) considered that restaurants/cafes were always or often helpful, 39% (*n* = 100/254) felt that they were ‘sometimes’ helpful, and 21% (*n* = 54/254) thought that they were rarely or never helpful. 

Forty-four per cent (*n* = 110/252) of respondents said that they had experienced restaurants refusing to prepare alternative foods; 44% (*n* = 110/252) said that they had not been allowed to eat their own prepared food in a restaurant; and 46% (*n* = 115/252) said that a restaurant had refused to cook low-protein pasta, burger mix or pizzas. The lack of low-protein food choices and inflexibility was considered unhelpful.

### 3.8. Changes That Would Encourage People with PKU to Dine Out

Seventy-nine per cent (*n* = 200/254) of respondents said changes would help improve their experience dining outside the home but 21% (*n* = 54/254) said changes would not help. There were 200 free text responses. The main themes are shown below and illustrated through a selection of verbatim quotes in Table 6.

### 3.9. Emotions around Dining Out

Respondents’ feelings and emotions before dining out are presented in Table 7.

When leaving a restaurant/café, only 35% (*n* = 88/254) of respondents said they were satisfied, with only 31% (*n* = 79/252) saying they were happy. Twenty-eight per cent (*n* = 71/254) left disappointed, 26% (*n* = 66/254) frustrated and 22% (*n* = 57/254) were still hungry. Adults with PKU (*n* = 43/118, 36%) were more than twice as likely to feel frustrated post-meal than caregivers of children under the age of 18 years (*n* = 23/136, 17%).

## 4. Discussion

This research is the first to purposefully investigate the eating out experiences, behaviours and concerns of people with PKU or their caregivers. Although eating out is a routine activity enjoyed by the general population, people with PKU chose not to do this regularly. While it is expected that people dining outside the home should derive social and psychological enjoyment [12], with satisfaction of appetite, and respite from low-protein meal preparation, our results suggest that people with PKU or their caregivers were unable to enjoy stress-free and spontaneous meals. In fact, 40% said eating out was associated with anxiety, only 9% derived any pleasure from it, with over one quarter of survey participants leaving restaurants feeling frustrated, disappointed, and still hungry. 

Individuals with PKU or their caregivers were eager to find restaurants that were willing to accommodate their dietary needs. Personalisation of menu choices with unlimited access to vegetables was considered almost mandatory for people with PKU. They commonly favoured familiar, non-chain/independent eating out venues that they had visited previously, with a proven track-record of preparing appropriate low-protein foods. Most preferred restaurants who cooked with fresh ingredients onsite rather than those who used pre-assembled meals that could not be modified. Some used eating establishments that had ‘build-your-own options’ (e.g., brands such as Subway or salad bars) allowing for more customization. Many found food-chain restaurants inflexible scoring disappointingly when rated by people with PKU or their caregivers. Restaurants often used pre-prepared foods, with some vegetable options being coated in wheat flour. Although vegan meal choices are now common in restaurants, they are usually high in protein.

Overall, incompatibility of menu choice with low-protein diets, inadequate food choice, uncertainty about the protein content of meals, and limited suitable drink options were all concerns of people with PKU or their caregivers. Consumers with PKU need transparency around meal ingredients, protein content and food portion size. Some restaurants only sell aspartame-containing soft drinks to avoid extra costs associated with sugar taxes. There was frustration that some restaurants would not agree to cook or even allow people with PKU to eat their own special low-protein foods e.g., low-protein bread, pasta and pizza bases prescribed by their general practitioner on their premises, even though the restaurant staff were unable to supply these foods themselves. Although some restaurants could offer gluten-free equivalents, these foods were often too high in protein for most people with PKU. 

Written information about the protein content of food provided on a website that could be studied in advance of a restaurant booking was considered helpful as it enabled the person with PKU or parents/caregivers to assess the suitability of food choices without the need for conversations with restaurant staff. Although most restaurants post their menus online, not all give their nutritional content and food portion sizes may differ if unweighted. Some fast-food chains post online the protein content of meals, but this information may be difficult to locate and given in small print tables. It was requested that restaurant food nutritional analysis and portion sizes should also be available by mobile app, with written reviews about special diet provision. There are currently no mandatory labelling requirements for any unpackaged products sold by catering businesses to state the protein content or list all the ingredients (except allergens, some additives and aspartame) [13]. The UK Government plans to introduce a new menu-labelling requirements law, which will enforce major foodservice operators to include a calorie count on the food items of both their digital and physical menus by April 2022, but it does not specify other nutrients or require provision of a full list of ingredients [14]. 

The results of this survey indicated that some people with PKU were reluctant to eat outside the home and experienced a spike in anxiety when visiting a restaurant because they anticipate it will not be a pleasurable experience. In another study on PKU, families reported avoiding eating out in restaurants, to prevent children from feeling excluded [15]. In our study, there was commonly social embarrassment, discomfort, and much sensitivity in the behaviours associated with social eating. The respondents experienced food worries about how others perceive them based on what they eat. To avoid causing others (e.g., staff or social companions) inconvenience, some respondents deliberately downplayed or did not mention their low-protein dietary requirements in conversations and opted for food options that were lower in protein and safe such as a baked potato, potato chips or a side salad. If they asked for alternative food choices, they felt that they were making unreasonable and excessive demands on staff. Some even felt they were being difficult when asking restaurant staff about the ingredients added to foods and the protein content of dishes. Others feared that the food venue would refuse to serve them after they had explained their dietary needs. Generally, people with PKU did not like drawing extra attention to their dietary needs within restaurants and any public discussions about their condition were commonly unwelcome.

The quality of the relationship or interaction that people with PKU or their parents/caregivers experience with food venues is important. They should be able to comfortably communicate with restaurant staff regarding their dietary needs. However, many perceive themselves as being made to feel as though they were a ‘fussy customer’ or a ‘nuisance’ so it constrained any conversation about food risks associated with incorrect food choices being served. Restaurant staff rarely proactively ask customers about special dietary needs, therefore leaving consumers to initiate any communication with staff regarding their requirements [16,17]. If the restaurant team genuinely listened to the dietary issues through taking the time to speak to the person and paying attention to what they said, the customer would be more forthcoming to discuss their dietary needs. This could lead to a willingness to modify food choices on a ‘plate’ in order to accommodate consumers’ needs and discretion whilst still holding conversations regarding dietary requirements. These actions are signs of extra care and respect. Commonly the waiter/waitress fail to understand the requests for low-protein food as there is no/low awareness of PKU, and people with PKU say ‘it is sometimes like talking to a brick wall’. The lack of knowledge leads to a customer perception of poor-quality provision. People with PKU might be more candid with staff whom they consider caring and trustworthy. The readiness of food establishments to adapt the dishes whilst respecting consumers’ food preferences and desire to try out different foods was also highly valued by patients with foods allergies [18,19]. 

A large proportion of the hospitality industry possess no or a very limited knowledge of special diets and may be unable to respond adequately to low-protein requests and this was clear from the results of the survey. However, ignorance of special diets by those people involved in delivering special dietary menus is not a defense for failing to meet the customer’s needs and expectations. Any current mandatory training predominantly focuses on food safety and technical preparation skills only, with an absence of education on special dietary requirements [20]. There should be mandatory special diet training for all employees who work in catering establishments. Special diet training has been shown to be effective. A short training programme on allergies was found to increase the knowledge and awareness of employees from all restaurants in one UK town as well as encouraging more information to be available for customers [21]. Furthermore, a survey that included 861 restaurant staff and members of the general public, found high levels of awareness of allergies and coeliac disease among trained chefs, in comparison to the general public and untrained staff, demonstrating the effectiveness of training [22]. 

### Limitations

Recruitment of participants for this online survey was via the NSPKU website and promoted on PKU social media sites, so respondents were limited to any individuals who had access to the internet using the appropriate technology. It is likely that respondents were people who accessed social media sites frequently, were not randomly selected, and the extent to which the sample matched the demographic characteristics of the general PKU population is unknown. However, the sample size was large, so this factor is likely to have had minimal impact on the overall results. In addition, caregivers acted as proxy respondents on behalf of children and described what they perceived to be their child’s feelings when eating out, so their answers may have been inaccurate. We did not distinguish between male and female respondents. Also, the number of respondents to scaled questions varied which may added errors to the results. Additionally, the questionnaire was non-validated, and the respondent’s level of understanding was unknown. Protein tolerance was not reported, and this may have influenced the respondents dining experiences. 

Furthermore, research to compare dining out experiences of patients with PKU and those with other conditions requiring dietary management may be useful to give additional insight into this practical issue.

## 5. Conclusions

In summary, there is a considerable lack of awareness and inability to successfully meet the needs of people with PKU on low-protein diets in restaurants and catering establishments in the UK. Reputation, revenue and customer relationships may be jeopardized if hospitality businesses do not meet the dietary needs of their customers. There is a need to better understand the knowledge and practices of restaurant and food-service establishment personnel toward the management of special diets in order to improve consumer experiences when eating out. Changes to staff training, flexibility to adapt menus, provision of more low-protein options, and a change in the law to enforce better availability of nutritional information in restaurants should be implemented. It is necessary to improve the experience of people with PKU and end the barriers they continually face in trying to enjoy a basic human social activity (dining out together) that most people can take for granted. 

## Figures and Tables

**Table 1:** Factors that prevent people with phenylketonuria (PKU) from eating out (*n* = 204) *.

Factors That Prevent People with PKU from Eating Out	Number of Responses *n* = 204	% Responses
Limited choice of suitable low protein foods	183	90
No information about the protein content of foods	136	67
Restaurant have limited knowledge about PKU	124	61
Feels like too much effort	108	53
Restaurants refuse to use low protein foods e.g., pasta	76	37
Embarrassed when explaining about PKU diet	69	34
The restaurant does not offer aspartame free drinks	60	29
Still feel hungry after eating out due to limited choice	60	29
Do not want to look different	56	28
Unhelpful restaurant staff	46	23
Have no choice but to eat foods that are not permitted	39	19
Restaurant staff often get my food order wrong	31	15
Other	22	11

* Multiple response question.

**Table 2:** Factors that influence the choice of restaurant/café when the person with PKU is eating out (*n* = 254) *.

Factors That Influence the Choice of Restaurant/Café When the Person with PKU is Eating Out	Number of Responses *n* = 254	% Responses
Good choice of low protein foods on the menu	186	73
Restaurant staff are happy to help	163	64
Catering staff will prepare a suitable meal independent of menu choice	121	48
Unlimited access to vegetables	120	47
Catering staff are happy to cook with low protein foods	103	41
Information about protein content of foods provided	102	40
Good choice of aspartame-free drinks	101	40
Like to socialize with family/friends regardless of food choice	80	32
Restaurant staff are discreet about the dietary needs for PKU	51	20
Restaurant staff have good knowledge about of the PKU diet	33	13
Other	17	7

* Multiple response question.

**Table 3:** What people with PKU normally do when eating out divided by age of respondents (*n* = 254).

Practices by Respondents	Respondents Aged < 18 Years	Respondents Aged ≥ 18 Years	Total
Just order from the menu and choose some-thing that may be suitable for PKU	64.7%	84.7%	74%
Ask the restaurant/cafe to prepare something different	8.8%	2.5%	5.9%
Bring in some pre-prepared low protein food from home	14.0%	1.7%	8.3%
Other	7.4%	8.5%	7.9%
Total responses	136	118	254

**Table 4:** Percentage of UK restaurant chains scored by adult patients or parents/caregivers of children with PKU scoring “good or very good” for their provision of low protein foods.

Restaurant	Number and % Who Scored Good or Very Good		Total Nunber of Answers for Each Restaurant
	*n*	%	Count
Hungry Horse	82	53%	154
Pizza Express	84	46%	181
McDonalds	84	46%	184
Wetherspoons	66	44%	149
Toby Carvery	31	39%	79
Las Iguanas	36	38%	94
Ask Italian	57	33%	173
Pizza Hut	48	29%	163
Wagamama	32	29%	112
Stonehouse Carvery	31	27%	114
Nandos	40	27%	149
Beefeater	25	26%	95
Chiquito	23	25%	93
Prezzo	19	24%	78
Frankie and Bennys	29	20%	144
Zizzi	19	20%	97
Bella Italia	20	18%	109
Harvester	20	18%	109
Greggs	13	18%	74
Brewers Fayre	13	15%	86
KFC	10	9%	107
Five Guys	14	8%	171
Café Rouge	13	7%	179
Giraffe	5	7%	72
Burger King	6	4%	155

**Table 5:** Open-ended responses to the questionnaire rating restaurant/café employee staff knowledge about PKU.

Theme	Examples of Verbatim Comments by Questionnaire Respondents
Low staff awareness of PKU (*n* = 68)	*‘most staff don’t even know what PKU is! When we explain it, many people seem to think we’re just being awkward for the sake of it.’* *‘the few times I tried to explain it, the waiter made fun of me and said I was ‘being picky.’*
PKU gets confused/conflated with food allergies or vegetarianism (*n* = 14)	*‘they get it confused with food allergies and some don’t even try to understand when we explain.’* *‘they just think you are a picky veggie/vegan.’*
Did not expect staff to be aware of PKU (*n* = 8)	*‘the employee cannot be expected to know about every condition.’* *‘I think it’s poor but the waitress should not need a medical exam to earn a minimum wage.’*
Staff rudeness/unhelpfulness (*n* = 8):	*‘nobody ever knows anything about PKU and people are sometimes very rude.’* *‘most of the time they think it’s made up and I’m being awkward.’*
Staff are sometimes helpful (*n* = 5):	*‘no one has ever heard of it, but some places are willing to try and make something work.’* *‘one time the restaurant did cook our own pizza base.’*
We do not discuss PKU in restaurants (*n* = 2)	*‘I would just find the experience not enjoyable if I had to keep asking questions about the menu.’*

Verbatim comments are presented in italic.

**Table 6:** Open ended responses to the questionnaire describing the changes that would help people with PKU dine out.

Theme	Examples of Verbatim Comments by Questionnaire Respondents
More low protein choices on the menu (*n* = 69)	*‘There should be at least one low protein menu choice that isn’t just vegetables and potato, with one or two flavour options (e.g., spices or sauce).’* *‘Cafes could offer soups, jacket potatoes or salads that don’t have added protein ingredients.’* *‘There should be more vegetarian options on the menu with the choice of exchanging ingredients such as low protein cheese and cream. Restaurants should also be able to cook e.g., low protein rice or pasta for people on a different diet.’* *‘Allow different ingredients on the menu to be mixed. For example, if mushrooms and grilled tomatoes are served on a steak—can they be bought as a portion on their own and served with a salad.’*
Educating and raising awareness amongst staff (catering/food retail) *n* = 64	*‘Staff more should be educated about PKU and adapt the restaurant menus.’* *‘Ensure the employees of restaurants know which foods contain protein and how important it is to have the nutritional information of their food readily available to customers.’* *‘I think it would help for people to know why phenylalanine/aspartame is often highlighted on drinks and why there is a need to stock aspartame free drinks—more so for smaller places like cafes.’* *‘Generally, more awareness is needed of PKU and the diet in the catering/dining sector as I think most staff now are prepared to accommodate dietary needs however simply don’t have the knowledge about it.’* *‘Training on how to specifically not make the customer feel like a nuisance via hospitality training.’* *‘An explanation that PKU is not an allergy.’* *‘Willingness to listen and not just say different foods cannot be used.’* *‘More understanding of very rare conditions—would they stop a blind person eating.’* *‘Have a card briefly explaining the details of the PKU diet for catering staff. I often find that they assume it’s like a peanut allergy’.* *‘All servers should ask all customers if anyone has any special dietary needs.’* *‘Restaurants should provide nutritional info about the food so customers can make informed decisions.’* *‘More friendly staff to make you feel confident and helpful.’* *‘Provide all staff with a fact sheet or information pack on what PKU is and what we can eat’*
Publishing protein content of menu items (*n* = 36)	*‘Having nutritional information in a booklet so anyone can read the protein level in different foods.’* *’Cafes and restaurants should list the amount of protein in their foods and drinks.’* *‘More nutritional info for sauces and vegan cheeses. Ability to get the chef to weigh foods too.’* *‘Nutritional information to be available for every dish offered on the menu so that people with PKU can make an informed choice about what they eat.’*
Staff should be able to adapt/tailor recipes (*n* = 15)	*‘Making a main meal up out of side dishes where choices are limited.’* *‘Restaurants more flexible in making meals with replacement ingredients to suit low protein diets.’* *‘Bring out the dish at the same time as other meals are being served so the person with PKU doesn’t feel singled out or different in anyway.’* *‘Being flexible with the menu’.* *‘Cooking something from scratch with suitable ingredients. Being happy to use prescription foods in their kitchen.’* *‘Talk about requirements away from table so child with PKU does not have to sit and listen to all the negotiations that have to go on before they can eat something.’*
Make it more normal/acceptable for people to bring their own food to be cooked (*n* = 11)	*‘They should cook something using your own pasta and rice.’* *‘Microwaves in food courts so can heat up our own food.’*
Publicise restaurants that are PKU friendly (*n* = 4)	*‘Give praise and positive reviews for the good restaurants, and shame those that have offered bad experiences.’* *‘More opportunities and advertising that they are happy to cater for special diets.’* *‘A sign on the restaurant to say we cater for all diets or be willing to help would be a start.’*

Verbatim comments are presented in italic.

**Table 7:** Emotions of adults with PKU/caregivers of children before dining out (n = 254) from multiple response question.

	Under 18 Years of Age		18 Years of Age or Over		Total Number of Respondents	
	*n*	%	*n*	%	*n*	%
Anxious	42	31%	59	50%	101	40%
Excited	54	40%	37	31%	91	36%
Hungry	37	27%	42	36%	79	31%
Happy	47	35%	29	25%	76	30%
Uneasy	32	24%	42	36%	74	29%
Concerned	22	16%	47	40%	69	27%
Pleasure	11	8%	12	10%	23	9%
Other	9	7%	11	9%	20	8%
Not applicable	12	9%	5	4%	17	7%
Total	136		118		254	

## Data Availability

The data will be made available from the authors upon reasonable request.

## Supplementary Materials

The following supporting information can be downloaded at: https://www.mdpi.com/article/10.3390/nu14030626/s1, Table S1: Full Questionnaire.
