# Supplementary material for: Hungry for Change: The Experiences of People with PKU, and Their Caregivers, When Eating Out
Source: Nutrients. 2022 Jan 31;14(3):626. doi: 10.3390/nu14030626 (PMC8839894; doi:10.3390/nu14030626)
Supplement: Supplementary file 1 [file nutrients-14-00626-s001.zip › nutrients-1538578-supplementary.pdf]

Supplementary material: Full Questionnaire

Title: Eating out in restaurants and cafes with Phenylketonuria (PKU)

1. Please select only one of the options below: (Please complete separate questionnaires if you have more than one child with PKU)

- I am an adult (18 years and over) with PKU
- I am a parent/carer of an adult with PKU (18 years and over)
- I am a parent/carer of a child with PKU (under 18 years old)

2. How old is the person with PKU? (Tick only one answer)
    - 0-4y
    - 5-11y
    - 12-18y
    - 19-25y
    - 26-35y
    - 36-50y
    - Over 50y

3. How often does the person with PKU go out for food to a restaurant or cafe?
    - Never
    - Less than once per month
    - Once per month
    - Once per fortnight
    - Once per week
    - 2-3 times per week
    - 4-5 times per week
    - Everyday

4. Would the person with PKU like to eat out more often?
    - Yes
    - No

5. Which of the following factors prevent the person with PKU from eating out more often? (Please select all that apply)
    - Embarrassed when explaining about the PKU diet
    - Do not want to look different
    - Unhelpful restaurant staff
    - Restaurant have limited knowledge about PKU
    - Limited choice of suitable low protein foods
    - No information about the protein content of foods
    - Restaurants refuse to use the low protein foods e.g. pasta
    - Restaurant staff often get my food order wrong
    - The restaurant does not offer aspartame free drinks
    - Restaurants do not allow food labels to be checked
    - Feels like too much effort
    - Have no choice but to eat foods that are not permitted in the PKU diet
    - Still feel hungry after eating out due to limited choice
    - Other

5.a. If you selected Other, please specify:

6. Is the choice of restaurant when eating out influenced by the need for a low protein diet for the person with PKU?
    - Yes
    - No

    6.a Please explain this.

7. Which of the following factors influence the choice of restaurant/cafe when the person with PKU is eating out? (Please select all that apply)
    - Like to socialize with friends/family regardless of food choice
    - Restaurant staff are discreet about the dietary needs for PKU
    - Good choice of low protein foods on the menu
    - Catering staff will prepare a suitable meal independent of menu choice
    - Information about protein content of foods provided
    - Restaurant staff are happy to help
    - Good choice of aspartame-free drinks
    - Catering staff are happy to cook with low protein foods
    - Unlimited access to vegetables
    - Restaurant staff have a good knowledge of the PKU diet
    - Other

    7.a. If you selected Other, please specify:

8. How would you rate the following restaurants when catering for a low protein diet?

    8.1 Macdonalds
    - Very poor
    - Poor
    - Okay
    - Good
    - Very good
    - Not applicable

    8.2 Pizza Express
    - Very poor
    - Poor
    - Okay
    - Good
    - Very good
    - Not applicable

    8.3 Nandos
    - Very poor
    - Poor
    - Okay
    - Good

- Very good
- Not applicable

8.4 Zizzi
- Very poor
- Poor
- Okay
- Good
- Very good
- Not applicable

8.5 Wagamamma
- Very poor
- Poor
- Okay
- Good
- Very good
- Not applicable

8.6 Chiquito
- Very poor
- Poor
- Okay
- Good
- Very good
- Not applicable

8.7 Frankie and Benny's
- Very poor
- Poor
- Okay
- Good
- Very good
- Not applicable

8.8 Las Iguanas
- Very poor
- Poor
- Okay
- Good
- Very good
- Not applicable

8.9 Bella Italia
- Very poor
- Poor
- Okay
- Good
- Very good
- Not applicable

8.10 Harvester

- Very poor
- Poor
- Okay
- Good
- Very good
- Not applicable

8.11 Wetherspoons

- Very poor
- Poor
- Okay
- Good
- Very good
- Not applicable

8.12 Pizza Hut

- Very poor
- Poor
- Okay
- Good
- Very good
- Not applicable

8.13 ASK Italian

- Very poor
- Poor
- Okay
- Good
- Very good
- Not applicable

8.14 Greggs

- Very poor
- Poor
- Okay
- Good
- Very good
- Not applicable

8.15 Burger King

- Very poor
- Poor
- Okay
- Good
- Very good
- Not applicable

8.16 Five Guys

- Very poor
- Poor
- Okay
- Good
- Very good
- Not applicable

8.17 KFC
- Very poor
- Poor
- Okay
- Good
- Very good
- Not applicable

8.18 Café Rouge
- Very poor
- Poor
- Okay
- Good
- Very good
- Not applicable

8.19 Giraffe
- Very poor
- Poor
- Okay
- Good
- Very good
- Not applicable

8.20 Prezzo
- Very poor
- Poor
- Okay
- Good
- Very good
- Not applicable

8.21 Brewers Fayre
- Very poor
- Poor
- Okay
- Good
- Very good
- Not applicable

8.22 Stonehouse Carvery
- Very poor
- Poor

- Okay
- Good
- Very good
- Not applicable

8.23 Toby Carvery
- Very poor
- Poor
- Okay
- Good
- Very good
- Not applicable

8.24 Hungry Horse
- Very poor
- Poor
- Okay
- Good
- Very good
- Not applicable

8.25 Beefeater
- Very poor
- Poor
- Okay
- Good
- Very good
- Not applicable

9. What do you normally do when eating out?
   - Just order from the menu and choose something that may be suitable for PKU
   - Ask the restaurant/cafe to prepare something different
   - Bring in some pre-prepared low protein food from home
   - Bring in some low protein food from home and ask the restaurant/café to cook it
   - Other

   9.a. If you select Other, please specify:

   9.b. How much notice would you give them that you are coming?

10. How would you rate your overall experience when eating out with PKU in restaurants/cafes? (1 being extremely poor and 10 being extremely good).
    - 1
    - 2
    - 3
    - 4
    - 5

- 6
- 7
- 8
- 9
- 10

11. How would you rate employee about PKU at restaurants/cafe you have previously been to (1 being extremely poor and 10 being extremely good).
    - 1
    - 2
    - 3
    - 4
    - 5
    - 6
    - 7
    - 8
    - 9
    - 10

    11.a What are the reasons for this ranking?

12. In general, how helpful are restaurants/cafe in trying to find a solution to cater for PKU?
    - Always helpful
    - Often helpful
    - Sometimes
    - Rarely helpful
    - Never helpful
    - Not applicable

13. Have you had any particularly good experiences with a restaurant/cafe with respect to catering for the PKU diet?
    - Yes
    - No

    13.a Which restaurant/cafe was this? And what did they do? Please describe your experiences.

14. Have you had any particularly bad experiences with a restaurant/cafe with respect to catering for the PKU diet?
    - Yes
    - No

    14.a Which restaurant/café was this? And what did they do? Please describe your experiences.

15. Have you experienced any of the following in a restaurant as a result of the low protein diet used in PKU?

    15.1 Refused to prepare different food
    - Yes
    - No
    - Not applicable

    15.2 Not allowed to eat my own prepared food in restaurant
    - Yes
    - No
    - Not applicable

    15.3 Refused to cook low protein pasta, burger mix or pizzas
    - Yes
    - No
    - Not applicable

    15.a Please explain what happened, if you answered "yes" to question 15.

16. Are there any changes you fell could be made, which would encourage the person with PKU to eat out more?

    - Yes
    - No

    16.a Please describe these changes.

17. Please name your top two most helpful restaurants/cafe when catering for the PKU diet.

    17.a Please explain why you choose those restaurants/cafes in question 17.

18. Please name your top two least helpful restaurants/cafes when catering for the PKU diet.

    18.a Please explain why you choose those restaurants/cafes in question 18.

19. How does the person with PKU usually feel before entering a restaurant/cafe? (You can select multiple if applicable)

- Excited
- Pleasure
- Happy
- Uneasy
- Concerned
- Anxious
- Hungry
- Not applicable
- Other

19.a If you selected Other, please specify:

20. How does the person with PKU usually feel after leaving a restaurant/Cafe? (You can select multiple if applicable)
    - Excited
    - Pleased
    - Happy
    - Satisfied
    - Disappointed
    - Angry
    - Anxious
    - Hungry
    - Frustrated
    - Sad
    - Not applicable
    - Other

20.a. If you selected Other, please specify:

21. Are there any other comments you like to make about eating out at restaurants/cafes with PKU?
